# Involvement of Plant Stem Cells or Stem Cell-Like Cells in Dedifferentiation

**DOI:** 10.3389/fpls.2015.01028

**Published:** 2015-11-18

**Authors:** Fangwei Jiang, Zhenhua Feng, Hailiang Liu, Jian Zhu

**Affiliations:** ^1^Department of Molecular and Cell Biology, School of Life Science and Technology, Tongji University, Shanghai, China; ^2^Translational Center for Stem Cell Research, Tongji University School of Medicine, Tongji Hospital, Shanghai, China

**Keywords:** plant stem cell, dedifferentiation, epigenetic, callus, asexual reproduction

## Abstract

Dedifferentiation is the transformation of cells from a given differentiated state to a less differentiated or stem cell-like state. Stem cell-related genes play important roles in dedifferentiation, which exhibits similar histone modification and DNA methylation features to stem cell maintenance. Hence, stem cell-related factors possibly synergistically function to provide a specific niche beneficial to dedifferentiation. During callus formation in *Arabidopsis* petioles, cells adjacent to procambium cells (stem cell-like cells) are dedifferentiated and survive more easily than other cell types. This finding indicates that stem cells or stem cell-like cells may influence the dedifferentiating niche. In this paper, we provide a brief overview of stem cell maintenance and dedifferentiation regulation. We also summarize current knowledge of genetic and epigenetic mechanisms underlying the balance between differentiation and dedifferentiation. Furthermore, we discuss the correlation of stem cells or stem cell-like cells with dedifferentiation.

## Introduction

Stem cells are a major research hotspot because of their therapeutic potential in several applications, such as human tissue replacement and drug development (Heidstra and Sabatini, 2014; Morus et al., 2014). Stem cell activity affects the remarkable longevity and strong regeneration capacity of plants (Heidstra and Sabatini, 2014). Animal stem cells reside in stem cell niches, which produce signals that balance self-renewal (division) and differentiation by generating daughter cells to form new tissues. Plant stem cells are also maintained in specific niches, called meristems, which are organized structures involved in post-embryonic development (Aichinger et al., 2012; Sozzani and Iyer-Pascuzzi, 2014). Plant stem cells can divide and further differentiate into various tissues and organs or form a new plant under specific environmental conditions (specific hormone backgrounds; Galinha et al., 2009).

Plant stem cells are maintained in primary and secondary meristems. Primary meristems possess three parts: apical meristem (shoots and roots), intercalary meristem, and floral meristem. Secondary meristems comprise lateral meristems, which include cambium and phellogen, and traumatic (callus) meristem (Morus et al., 2014). Intercalary meristem is present between mature tissues in certain plants, does not differentiate with other adjacent meristems into mature tissues, and is silently preserved. However, intercalary meristem can divide and differentiate during a specific period of development or under specific environmental conditions (Beveridge et al., 2007). Numerous studies and reviews have focused on the stem cell behavior (Aichinger et al., 2012; Lee et al., 2013; Yadav et al., 2013; Heidstra and Sabatini, 2014).

Procambium/cambium cells satisfy the criteria for stem cells because of their capacity for long-term self-renewal and ability to differentiate into one or more specialized cell types; as such, these tissues serve as niches for plant stem cells (Alison et al., 2002). Several types of cells also possess stem cell properties. For instance, differentiated pericycle cells initiate lateral root formation after leaving the root meristem (Beeckman and De Smet, 2014). Sena et al. (2009) reported that stem cell-like properties of differentiated cells can mediate complete organ regeneration when plant root meristems are dispersed. These cells exhibit properties similar to those of stem cells and are thus defined as stem cell-like cells in this review.

Callus is predominantly formed from a pre-existing population of stem cells (Sugimoto et al., 2010, 2011) and from cell dedifferentiation. This presumption means that stem cells or stem cell-like cells play important roles in callus formation. Hence, stem cells or stem cell-like cells may be involved in dedifferentiation of other cells to a certain degree. Other studies also consider that regeneration or dedifferentiation does not require a functional stem cell niche in plants (Sena et al., 2009). Thus far, the relationship between stem cells and dedifferentiation remains unclear. In this study, we reviewed the mechanisms of stem cell niches and dedifferentiation in plant growth. We also analyzed the potential correlation between stem cells and plant cell dedifferentiation. This review provides insights for understanding plant growth and behavior.

## Dedifferentiation

Dedifferentiation is the transformation of cells from a given differentiated state to a less differentiated or stem cell-like state and leads to reacquisition of pluripotency; dedifferentiation is a cellular process associated with reentry into the cell cycle, trans/redifferentiation, or even cell death (Grafi, 2004). This process is commonly studied in plants and amphibians, zebrafish, and other basal life forms capable of organ regeneration (Brockes and Kumar, 2002; Poss et al., 2002; Jopling et al., 2010; Sugimoto et al., 2011; Xu and Huang, 2014). Cellular dedifferentiation mostly occurs in aged or damaged tissues for regeneration (Sanchez Alvarado and Tsonis, 2006).

Plant cellular dedifferentiation is manifested by transition of differentiated cells into protoplasts (Jamet et al., 1990; Zhao et al., 2001) and features widespread chromatin decondensation, similar to that of stem cells (Zhao et al., 2001; Tessadori et al., 2007; Ondrej et al., 2010). Auxin and cytokinin induce protoplasts to reenter the cell cycle, proliferate, and form callus (Grafi, 2004; Muraro et al., 2011). Stress conditions, including protoplasting (plant cell wall is degraded by enzymes), wounding, and exposure to dark or heat, induce somatic cells to change their fate via dedifferentiation (Malamy, 2005; Grafi et al., 2011a,b; Florentin et al., 2013; Feher, 2014). Researchers propose that the dedifferentiated state is a transient state of senescence because senescing cells share similar features, such as gene expression and chromatin modification, with dedifferentiating protoplast cells (Balazadeh et al., 2008; Damri et al., 2009; Yadav et al., 2009). This hypothesis indicates that dedifferentiation precedes cell death (Grafi, 2004). Thus, dedifferentiation may be involved in cell fate switch.

A study revealed that cell dedifferentiation in animals is triggered by signals from the stem cell niche and leads to dynamic cellular rearrangements. This process possibly involves Janus kinase–signal transducer and activator of transcription signaling pathway because its downregulation leads to inhibition of dedifferentiation (Sheng et al., 2009). In addition, epimorphosis requires preexisting stem cells or dedifferentiation-generated progenitor cells to proliferate, differentiate, and replace lost cells (Cai et al., 2007). These findings support that plant stem cells and/or stem cell niches are significantly associated with dedifferentiation.

## Involvement of Stem Cell-Related Genes in Dedifferentiation

Dedifferentiation is a complex process related to the expression and regulation of specific genes. Several studies investigated the relationship between genes and dedifferentiation (Berdasco et al., 2008). Several lines of evidence support the hypothesis that stem cell-related genes are involved in dedifferentiation (Yadav et al., 2010; Jopling et al., 2011).

Our previous studies showed that stem cells initially remain in the vascular bundle and divide, and callus is formed after petiole explants are cultured for 24–36 h (Yu et al., 2010; Li et al., 2011a). The mRNA chips were analyzed to screen related genes. The results showed that *WUSCHEL* (*WUS*) and *NO APICAL MERISTEM* (*NAM*) gene families are strongly expressed in petiole dedifferentiation (Liu et al., 2010). Other studies also reported that *WUS* is highly expressed in several callus lines (Iwase et al., 2011a), and overexpressing this gene forms callus and somatic embryos (Zuo et al., 2002). Moreover, increased *WUS* levels lead to dedifferentiation of stem cell progenitors into stem cells (Reddy and Meyerowitz, 2005; Yadav et al., 2010). *WUS* is a stem cell niche signal important to maintain stem cells in a relatively undifferentiated state (Laux et al., 1996; Mayer et al., 1998; Yadav et al., 2013; Zhou et al., 2015). Thus, *WUS*, a well-known stem cell-related gene, is involved in dedifferentiation. Similarly, teosinte branched1/Cycloidea/proliferating cell factor is a transcriptional activator that negatively regulates shoot meristem maintenance (Koyama et al., 2010) and cell dedifferentiation (Ikeda and Ohme-Takagi, 2014). Overexpressing stem cell-related genes, including master regulators in egg cell fate (*RKD1* and *RKD2*, Koszegi et al., 2011) and embryonic fate (*RKD4*, *LEC1*, *LEC2*, *AGL15*, and *BBM*, Srinivasan et al., 2007; Thakare et al., 2008; Waki et al., 2011; Guo et al., 2013), is sufficient to induce callus formation (Ikeuchi et al., 2013). Most LATERAL ORGAN BOUNDARIES DOMAIN (LBD) transcription factors are involved in a regulatory loop that maintains shoot meristem and defines the lateral organ boundary (Bell et al., 2012). For instance, LBD15 is involved in shoot apical meristem development by regulating *WUS* expression (Sun et al., 2013). As such, numerous *LBD* genes are possibly involved in dedifferentiation (Liu et al., 2010). Furthermore, *LBDs* directly form callus in *Arabidopsis* regeneration (Fan et al., 2012). In rice, *ADVENTITIOUS ROOTLESS1* (*ARL1*), an *LBD* family member, is involved in hormone-mediated pericycle cell dedifferentiation and promotes initial cell division (Liu et al., 2005). These findings show that stem cell-related genes play an important role in dedifferentiation. Hence, we presume that dedifferentiation may share a similar regulatory mechanism with the stem cell niche.

The AP2/ERF transcription factor WOUND INDUCED DEDIFFERENTIATION 1 (WIND1) and its close homologs, including WIND2 to WIND4, induce wounding and promote cell dedifferentiation in *Arabidopsis* (Iwase et al., 2011b). A similar homologous gene, namely, *ThWIND1-L*, was found in *Thellungiella halophila* (Zhou et al., 2012). However, the direct relationship of *WINDs* to stem cell niche remains inconclusive. WIND activates cytokinin signaling but not auxin signaling, whereas auxin alone, not cytokinin alone, can induce callus formation (Li et al., 2011a). As such, dedifferentiation may involve several pathways comprising stem cell-related genes. The dedifferentiation mechanism is not a precise copy of the regulatory mechanism in a stem cell niche. Therefore, numerous genes regulate one phenomenon by different pathways and coordinate with each other to maintain a specific niche. The balance in niches can decide the cell fate and facilitates plant growth, development, asexual reproduction, and pluripotency. This phenomenon is represented in a “seesaw model,” which posits that the reprogramming of animal cells is affected by the balance in interactions among genes (Shu et al., 2013).

The types and levels of cell differentiation differ in explants. Specific cells, such as differentiated cells, switch fate during dedifferentiation, whereas other cells, such as stem cells, are not affected by differentiation. However, not all parenchymal cells in explants can reach a stem cell-like status because some of these cells may die. Hence, when the explants encounters a cell fate decision, a certain signal should indicate which cells should survive. This signal may be secreted by the cell itself to determine autonomous events in each cell. Moreover, signal communication may exhibit similar characteristics to the mode used by stem cells to decide their number in the microenvironment. In several cases and in organisms ranging from bacteria to humans, cells adopt a particular fate stochastically without apparent regard to the environment or history (Losick and Desplan, 2008). In the large majority of cases, cells acquire their fate by virtue of lineage and/or proximity to an inductive signal from another cell (Losick and Desplan, 2008). Signals exchanged between neighboring cells, similar to the Notch receptor in animals, can amplify and consolidate molecular differences, which eventually dictate cell fates (Artavanis-Tsakonas et al., 1999; Drevon and Jaffredo, 2014). Limited direct evidence confirms that the cell–cell communication plays an important role in dedifferentiation. However, cell-to-cell transport through plasmodesmata was detected in tree callus (Pina et al., 2009). We assume that the signal from another cell also plays an important role in callus formation and may exhibit similar characteristics to the signal used by stem cells to decide their number in the microenvironment. Communication is a fundamental mechanism for coordinating developmental and physiological events in multicellular organisms. This process is also widely shared as a key mechanism in a stem cell niche (Geiger and Van Zant, 2002; Oatley and Brinster, 2012). For instance, heterotrimeric G proteins are key molecules that transmit extracellular signals. The G protein beta-subunit1 AGB1 and RPK2, a major CLV3 peptide hormone receptor, work synergistically in stem cell homeostasis through physical interactions to facilitate meristem development (Ishida et al., 2014). Communication in dedifferentiation may be the next significant study area.

## Similar Epigenetic Modifications Shared in Stem Cell Maintenance and Cellular Dedifferentiation

Dedifferentiation is controlled by various epigenetic mechanisms, including chromatin structural changes, as well as DNA and gene methylation. Epigenetic changes in stem cells are reversible and provide plasticity in plants; plasticity allows differentiated cells to recover totipotency under certain physiological and environmental conditions.

Open chromatin conformation characterizes dedifferentiated cells in plants and animals (Grafi, 2004; Meshorer and Misteli, 2006; Gaspar-Maia et al., 2011; Grafi et al., 2011b). This characteristic is necessary to maintain stem cell developmental capacities, including self-renewal and differentiation into multiple cell types (Melcer and Meshorer, 2010; Gaspar-Maia et al., 2011). To support transcriptional promiscuity, chromatin in stem cells is “plastic” or “open,” with a decondensed heterochromatin architecture, enriched active histone modifications, and hyperdynamic association of chromatin proteins with chromatin (Zipori, 2004; Meshorer and Misteli, 2006). The overexpression of genes involved in chromatin modification pathways in stem cells confirms the existence of flexible chromatin structures in shoot apical meristems (Yadav et al., 2009).

Similar to stem cells, dedifferentiating plant cells acquire an open, decondensed chromatin architecture (Zhao et al., 2001; Meshorer and Misteli, 2006; Tessadori et al., 2007), which is essential but not sufficient to initiate gene transcription. In *Arabidopsis* protoplasts, the acquisition of pluripotency is associated with chromatin reorganization in specific domains (Jiang et al., 2013). Grafi et al. (2007) demonstrated that histone methylation is required to establish the dedifferentiated state.

In mammals, DNA methyltransferase 1 (DNMT1) is responsible for self-renewal and differentiation of stem cells, as well as self-renewal of mammalian somatic tissues (Chan et al., 2005; Sen et al., 2010). Similar to DNMT1, MET1 is a methyltransferase involved in maintaining DNA methylation in *Arabidopsis* (Chan et al., 2005). Root explants with *MET1* mutants exhibit reduced callus formation in culture with 2,4-D (analog of auxin); this finding indicates that DNMT1-like methyltransferases are related to dedifferentiation process-callus formation (Berdasco et al., 2008). Active DNA demethylation in plants is generally performed through a base excision repair pathway, which can be induced following the activity of the DME/ROS1 family of DNA glycosylases or by the coupled activities of 5-methylcytosine deaminase (Zhu, 2009; Zhang and Zhu, 2012).

In cooperation with DNA methylation, histone modifications regulate gene expression by altering chromatin structure and transcriptional activity, thereby influencing callus formation (Desvoyes et al., 2010). KRYPTONITE (*KYP*)*/SUVH4* mutants, which encode a histone H3 lysine 9 (H3K9) methyltransferase, produces defects during stem cell proliferation and callus formation (Grafi et al., 2007). Moreover, *MET1* and *KYP* mutants alter the expression of *WUS*, a marker for plant stem-like cells (Li et al., 2011b). These findings support that histone methylation activity is required to establish/maintain the dedifferentiated state and/or re-entry into the cell cycle, as well as callus formation, in plant cells (Berdasco et al., 2008).

The promoter activity of stress-induced *ANAC2* [*abscisic acid-responsive NAC* (*NAM/ATAF1*/*2*/*CUC2*), also known as *Arabidopsis* transcription activation factor (*ATAF1*)] is associated with widespread decondensation of pericentric chromatin (Florentin et al., 2013); the *ANAC2* promoter is also meristem specific (Damri et al., 2009). The promoter of *ANAC2* activity is upregulated in dedifferentiating protoplast cells and in cells subjected to various biotic and abiotic stress conditions (Damri et al., 2009; Yadav et al., 2009; Florentin et al., 2013).

The *ANAC2* promoter is active not only in meristems but also in leaf primordia and young leaves (Florentin et al., 2013). Thus, stem cell state may be maintained in plant tissues and organs during the early development stages in the form of stem cell-like cells (Laux, 2003). In humans, stem cells may stimulate paracrine effects to orchestrate wound healing and cancer progression (Dittmer and Leyh, 2014). These stem cells may secrete growth factors or cytokines, as well as non-protein factors, such as RNAs and lipids, to communicate with other cells. Plant stem cells may also produce factors to regulate hormones in a paracrine effect in response to wound or other stresses. Thus, the widely retained stem cells or stem cell-like cells may give rise to dedifferentiation. However, whether stem cells assist neighboring cells to alter gene expression and influence dedifferentiation process require further research.

## Stem Cells or Stem Cell-Like Cells Involved in the Dedifferentiation Process

Asexual reproduction maintains the characteristics of species in a relatively stable environment and is considered dedifferentiation. *Kalanchoe daigremontiana* produces viviparous seedlings to accomplish asexual reproduction (Garces et al., 2014). Some retained initiating cells located in the nicks of mature leaf edges can divide and differentiate into viviparous seedlings (Garces et al., 2007). The young plants of *Graptopetalum paraguayense* and *Crassula portulacea* originate from a wound in the leaf. The young plant of *G. paraguayense* is only generated at the proximal petiole, in which several layers of stem cell-like cells are located; conversely, the adventitious buds of *C. portulacea* are produced by the activities of procambium cells (stem cell-like cells) in the vascular bundle in any wound of a leaf. These observations imply that the retained stem cells or stem cell-like cells may initiate asexual reproduction of plants (Murashige, 1974).

During callus formation in petiole explants, cells located near vascular tissues and are adjacent to procambium cells (stem cell-like cells), more easily survive than other cells (Bongso and Richards, 2004; Yu et al., 2010). Pre-existing stem cells or stem cell-like cells may produce signals that promote cell dedifferentiation through cell-to-cell communication. However, direct evidence of cell-to-cell communication remains insufficient. In addition, the dedifferentiation activities of cells may be determined by their cell types, age, or other features.

In *Drosophila*, airway stem cells can influence the dedifferentiation of airway epithelial cells into stable and functional basal stem cells *in vivo* and *ex vivo* (Tata et al., 2013). These findings suggest that stem cells and their progeny can reciprocally modulate one another through a precise and local control mechanism. This premise supports that plant stem cells/stem cell-like cells perform a significant function in dedifferentiation.

## Conclusion

Significant advances have been achieved in stem cell research. Accumulating evidence suggests that a complicated regulatory network contributes to modulated plant stem cell maintenance, although the detailed mechanisms have not been completely revealed. This complicated regulatory network may include genes, hormones, and epigenetic factors, which play significant roles in dedifferentiation. These factors build and maintain a specific niche. In addition, stem cells or stem cell-like cells may influence the dedifferentiating niche. Studies documented that stem cells retained in the tissues contribute to callus formation under certain environmental conditions. Moreover, plant cells that underwent stress or senescence present a dedifferentiation patter similar to the stem cell-like state prior to entry into new cell fates. Stem cells may communicate with neighboring cells by releasing specific signals during dedifferentiation of neighboring cells. This communication process may be enhanced by adding stem cells. However, further research must be performed to confirm this assumption. Elucidating signals/factors involved in the governing dedifferentiating niche, including stem cells, can provide insights into designing sophisticated and specific molecular tools to systematically manipulate organ regeneration.

### Conflict of Interest Statement

The authors declare that the research was conducted in the absence of any commercial or financial relationships that could be construed as a potential conflict of interest.
